# Spatial localization of the first and last enzymes effectively connects active metabolic pathways in bacteria

**DOI:** 10.1186/s12918-014-0131-1

**Published:** 2014-12-14

**Authors:** Pablo Meyer, Guillermo Cecchi, Gustavo Stolovitzky

**Affiliations:** IBM Computational Biology Center, Yorktown Heights, NY USA

**Keywords:** Metabolism, Enzyme localization, Fluorescent imaging, Metabolic network, Enzymatic activity, Metabolic pathways, Bacteria

## Abstract

**Background:**

Although much is understood about the enzymatic cascades that underlie cellular biosynthesis, comparatively little is known about the rules that determine their cellular organization. We performed a detailed analysis of the localization of *E.coli* GFP-tagged enzymes for cells growing exponentially.

**Results:**

We found that out of 857 globular enzymes, at least 219 have a discrete punctuate localization in the cytoplasm and catalyze the first or the last reaction in 60% of biosynthetic pathways. A graph-theoretic analysis of *E.coli’s* metabolic network shows that localized enzymes, in contrast to non-localized ones, form a tree-like hierarchical structure, have a higher within-group connectivity, and are traversed by a higher number of feed-forward and feedback loops than their non-localized counterparts. A Gene Ontology analysis of these enzymes reveals an enrichment of terms related to essential metabolic functions in growing cells. Given that these findings suggest a distinct metabolic role for localization, we studied the dynamics of cellular localization of the cell wall synthesizing enzymes in *B. subtilis* and found that enzymes localize during exponential growth but not during stationary growth.

**Conclusions:**

We conclude that active biochemical pathways inside the cytoplasm are organized spatially following a rule where their first or their last enzymes localize to effectively connect the different active pathways and thus could reflect the activity state of the cell’s metabolic network.

**Electronic supplementary material:**

The online version of this article (doi:10.1186/s12918-014-0131-1) contains supplementary material, which is available to authorized users.

## Background

Recent advances in bacterial biology have shown that cells contain a highly organized cytoplasm composed of cytoskeleton and internal compartments [1]. The image of a cell as a “bag of enzymes” has given way to a view where molecules and proteins localize at the right time in the right place in order to perform their necessary functions; however, enzymes involved in the most basic metabolic functions are generally thought to be freely diffusing in the cytoplasm. An exception is the well studied chemotaxis pathway, where advanced microscopic techniques have revealed that proteins self-assemble stochastically in clusters whose function has only been theorized [2,3]. Conversely, the biochemical reactions that underlie metabolic functions in cells are well understood [4], and have been reproduced *in vitro* [5,6]; nevertheless, aside from a few exceptions where enzymes form macromolecular complexes [7-9], little is known about the cellular organization of enzymes.

It is possible that the existence of large multi-enzyme complexes, as opposed to freely diffusing enzymes, could either be determined by constraints limited to highly specialized reactions, or a general mechanism used throughout the cell to achieve a generic metabolic function. Supporting the latter option, the hypothesis of metabolic channeling proposes that reaction products in a metabolic pathway move from one active site to another within tightly associated multi-enzyme complexes. Such organization may possess several kinetic advantages for the cell [10,11]; for instance, this compact geometry could prevent metabolic intermediates from diffusing away (i.e. substrate channeling), or increase the metabolic flux through the pathway. Several examples such as the asparagine and tryptophan synthases [12,13], carbon fixation enzymes [7,14], polyketide synthases [15] or the porphyrin [16] and phycoerythrobilin [17] synthesis pathways support the existence of substrate channeling and formation of multi-enzyme complexes in bacteria. One major caveat of the metabolic channeling hypothesis is the inherent rigidity of a multi-enzymatic complex, making any mutation dissociating parts of the tight complex potentially prone to reduce or eliminate its enzymatic activity.

In order to determine if enzyme localization is a generic and functionally relevant property of enzymatic reactions in cells, we studied the distribution of genome-wide GFP-tagged enzymes in *E.coli* cells growing exponentially. We found that 25% of known cytoplasmic enzymes show a discrete punctuate localization in the cytoplasm. When the *E.coli* biosynthetic pathways were organized into Elementary Flux Patterns (EFPs), we observed a significant preference for the first or last enzyme in such EFP-defined pathways to be localized and that localized enzymes form a tree-like hierarchical structure with higher within-group connectivity.

## Results

### *E.coli* enzymes in the first and last position of pathways preferentially localize to cytoplasmic foci

**Figure 1:**
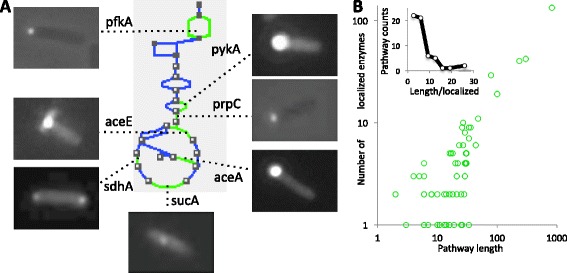
**Localization of enzymes in** ***E. coli*** **pathways. A**. Images of localized enzymes from glycolysis and TCA cycle. In the diagram, indicated in green are the reactions for which the associated enzyme localizes and in blue reactions where enzymes are diffuse. Triangle indicates *amino-acids*, square *carbohydrates*, circles for *other metabolites.* **B**. Log-log plot of the number of localized enzymes against the number of reactions in a pathway – pathway length– for the 59 pathways showing localized enzymes (Pearson correlation is 0.98). *Inside.* Distribution of the ratio of pathway length to number of localized enzymes, mean value is 7.1.

The complex organization of the bacterial cytoplasm suggests that enzymes could, not only freely diffuse, but also show spatial ordering in the cytoplasm. To explore the rules of putative enzymatic organization, we took advantage of the images of *A complete Set of E.coli K-12 ORF Archive* (ASKA) library of GFP-tagged ORF clones [18] displayed on the GenoBase database to study the cytoplasmic distribution of *E.coli* biosynthetic enzymes. We used the images displayed on GenoBase representing *E.coli* cultures growing exponentially and expressing each a protein whose C-terminus was fused to a GFP (see *Methods*). The putative introduction of localization artifacts by GFP-tags [19] was minimized by restricting our analysis to globular enzymes, excluding membrane-bound proteins, and by carefully chosing controls for the statistical analysis. Based on the localization classification shown in GenoBase we compiled the presence of 219 enzymes in the list of 516 non-membrane bound fusions that showed GFP focal distribution (see *Sup. Mat* for a complete definition of the list). As the KEGG collection, a set of manually drawn pathway maps indicating molecular interactions and reactions networks, indicates that *E.coli* K-12 MG1655 has 857 enzymes involved in known metabolic reactions, at least 25% of the enzymes in *E.coli* cells growing exponentially show a discrete punctuate localization at diverse locations of the cytoplasm (see Figure 1A & Additional file 1: Figure S1). Also, from the 86 *E.coli* metabolic pathways defined in KEGG, 59 showed at least one enzyme localized and the number of localized enzymes scaled linearly with pathway length (see Figure 1B, R^2^ = 0.98).

**Figure 2:**
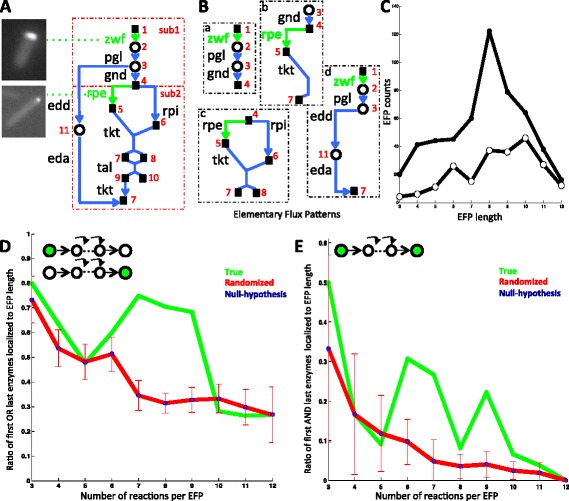
**Localization of first and last enzymes in** ***E.coli*** **EFPs. A**. pentose phosphate pathway (PPP) is used to illustrate the construction of elementary flux patterns from *E.coli* biochemical pathways. Localized enzymes and their associated images are shown in green. Two subsystems are considered –sub1 and sub2– and correspond to the oxidative and reversible parts of the PPP. **B**. These subsystems lead to four EFPs. The end-product of EFPs *b, c, d,* are metabolites that feed into glycolysis. Enzymes are indicated by blue arrows with their associated gene names. Localized enzymes are shown in green and diffuse enzymes in blue. Black squares are *carbohydrates* and circles indicate *other metabolites*, both identified by numbers in the legend. : 1. Glucose-6-phosphate 2. 6-phosphogluconolactone 3. 6-phosphogluconate 4. ribulose-5-phosphate 5. xylulose- 5-phosphate 6. ribose-5-phosphate 7. Glyceraldehyde-3-phosphate 8. Sedoheptulose- 7-phosphate 9. Erythrose-4-phosphate 10. Fructose-6-phosphate 11. 2-Dehydro-3-deoxy gluconate-6-phosphate. **C**. Distribution of EFP lengths when at least one enzyme localized (full dots) and when more than one enzyme localized (open dots). **D**. Ratio that first OR last enzyme are localized to number of Elementary Flux Patterns (EFP) for lengths of 3 to 12 reactions (green), calculated ratio for randomly chosen enzymes (blue) and by randomization of localized enzymes (red). Dotted arrows represent pathways 1&2 connected via localized reactions **E**. Ratio that first AND last enzyme are localized to EFP length (green), calculated ratio for randomly chosen enzymes (blue) and by randomization of localized enzymes (red).

Due to the high connectivity of metabolic reactions, it is difficult to uniquely assign pathway positions to enzymes. We decided to analyze the position of localized enzymes organizing biochemical pathways from a genome-scale metabolic network of *E. coli* [20] into Elementary Flux Patterns (EFP) [21]. EFPs are sets of reactions defining balanced physiological fluxes through a particular subsystem of metabolism and correspond to basic metabolic routes [22]. EFPs define the intersection of biological and flux related properties of pathways. For example we show in Figure 2 how the Pentose Phosphate Pathway (PPP) would be decomposed into four EFPs, based on the definition of two sub-systems corresponding to the oxidative and reversible part of the PPP.

We considered 725 *E.coli* EFPs composed of 3 to 12 reactions per EFP, from 27 different biochemical pathways including nucleotide, amino acid, nitrogen, central and alternate carbon metabolism, as defined in [21]. 529 EFPs had at least one enzyme localized, 126 reactions were catalyzed by localized enzymes in the first position of the EFP, 226 by localized enzymes in the last EFP position and 228 with at least one localized enzyme in an intermediary position. Finally 33 EFPs showed the first and the last enzymes to be localized (see Additional file 2: Table S1 & Figure 2 for EFP details). In order to obtain an estimate of the over-representation of localized enzymes in the first and last positions of the 529 EFPs, we computed for a given EFP length, the ratio of the number of EFPs having the first *or* the last enzyme localized to the total number of same-length EFPs having at least one enzyme localized (Figure 2D green line). We then compared this value to a null hypothesis consisting of the same ratio now computed on each of the 529 EFPs having the position of the localized enzymes permutated 10000 times at random (Figure 2D red line). We also calculated the ratio that randomly chosen enzymes localize in the first or last step (see *Methods* and plotted in Figure 2D blue). Figure 2D shows that for pathways composed of 3 to 9 reactions, the ratio of localizing enzymes in the first or last position is higher than 50%. The p-values associated to the null distribution confirm this observation, excepting reactions of length 5 (see Additional file 3: Table S2). Interestingly, a plateau of significant high localization ratio values is reached for longer pathways composed of 7–9 reactions.

We likewise computed for the subset of 222 EFPs that had two or more localized enzymes, the ratio of having the first *and* the last enzymes localized to the number of reactions for a given EFP length (Figure 2E green line) and generated the null hypothesis distributions by randomization of EFPs (Figure 2E red line) or by calculating the ratio that randomly chosen enzymes localize in the first and last step (described in *Methods* and plotted in Figure 2E blue). Figure 2E shows that for intermediate pathways composed of 6 to 9 reactions, the ratio of localizing enzymes in the first or last position is high. Note that EFPs having 3–5 reactions are just by chance prone to show enzyme localization in the first and last reaction and hence the p-values were not significant (see Additional file 3: Table S2). Interestingly, the most significant ratio was reached for pathways composed of 7–9 reactions, an interval including the number of mean localized enzymes per pathway (Figure 1B inset).

Around 60% of EFPs (319 out of 529) have either the first or the last enzyme localized and 33 EFPs have both enzymes localized. This indicates an important role for the start or end position of pathways that might underlie enzymatic regulatory strategies.

### Localized enzymes form a tree-like sub-network of highly interconnected nodes in *E.coli*’s metabolic network

**Figure 3:**
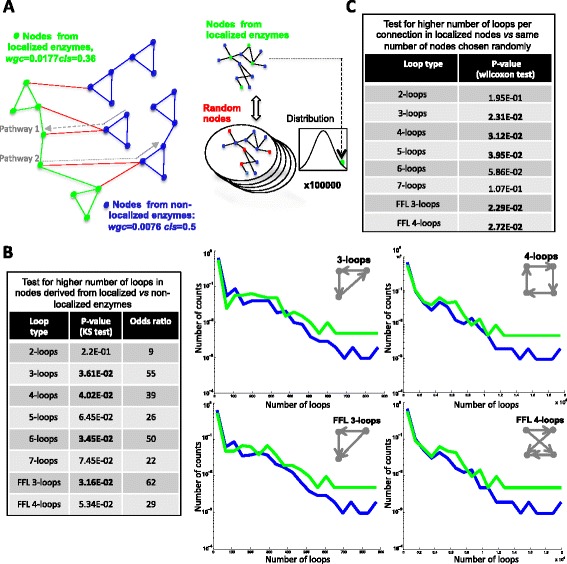
**Localized enzymes form a tree-like loop-dense network** A matrix representing the connectivity of the 1214 different biochemical reactions of *E.coli* was constructed (see Additional file 1: Figure S1B). **A**. *Left* Diagram illustrating the clustering (cls) and within-group connectivity (wgc) of localized (green) and non-localized (blue) enzymes. Red edges represent inter-group connections. Dotted arrows represent pathways 1 & 2 connected via localized reactions. *Right* Diagram illustrates the distribution of randomly selected nodes constructed to compare mean loop density with nodes from localized enzymes. **B**. *Left.* Associated p-values of the KS-test for higher number of loops in nodes derived from localized enzymes compared to non- localized ones. *Right.* Four plots showing the Log-scale distributions of the number of loops for each node in localized (green) and non-localized (blue) enzymes. The type of loop is indicated *top right*. **C**. Table shows the associated p-values for higher number of loops per connection in nodes derived from localized enzymes compared to nodes chosen randomly.

Enzyme localization is present in most pathways of the *E.coli* metabolic network (Figure 1B & Additional file 1: Figure S1) and is prevalent in the first and last position (Figure 2D). In order to explore the properties of these localized enzymes when considering the complete *E.coli* set of metabolic reactions, we constructed a metabolic network using the KEGG definitions of compounds, reactions and enzymes (see *Methods**)*. The metabolic network is described by an asymmetric Boolean square matrix *Mij* of 1214 unique reactions where each reaction is associated to an enzyme and a non-zero matrix value *mij = 1* indicates that the reaction *i* feeds into reaction *j* by producing a compound used by reaction *j* (see Additional file 4: Figure S2A). While trying to differentiate nodes of 189 reactions catalyzed by localized enzymes from 1025 others, we plotted for each node the number of outcoming reactions against the number of incoming reactions (Additional file 4: Figure S2B). Localized enzymes were found at low and high-connected nodes varying from 1 to 50, but no significant differences were found in the distributions of incoming or outcoming reactions, nor in length of shortest paths, when considering localized and non-localized enzymes (Additional file 4: Figure S2B insets). However, when the two classes where considered separately (Figure 3A left & Additional file 4: Figure S2B green and blue), we observed that localized reactions have a significantly higher probability to establish within-group connections (wgc) than non-localized ones have (0.0177 vs 0.0076 t-test z-score = 9.32 for a distribution choosing random nodes as shown in the right diagram of Figure 3A). Interestingly, this higher within-group connectivity does not result in higher within-group structure as localized reactions form less triangulations among themselves than localized ones, hence displaying a more tree-like hierarchical structure of connections (see green nodes left diagram Figure 3A and Additional file 4: Figure S2A). Triangulations were measured by the clustering coefficient, yielding 0.36 vs 0.5 for localized and non-localized reactions respectively (Kolmogorov-Smirnov, KS test. p-value < 1E-12, see *Methods* for details on scaffold triangulations for clustering coefficient).

### Localized enzymes are associated to loop-dense nodes in *E.coli*’s metabolic network

We next quantified the number of n-step loops encompassing each reaction in the full connectivity matrix, in order to further characterize the network structure with a measure of functional relevance [23]. For reaction *i* this is given by [*M*^*n*^]_*ii*_, the diagonal value of the matrix raised to the *nth* power. Note that, unlike the computation of the clustering coefficient, here we considered the direction of connections, so that feedback and feed-forward loops can be distinguished. In Figure 3B we compared the distributions of [*M*^*n*^]_*ii*_ 2 ≤ n ≤ 7 for reactions catalyzed by localized and non-localized enzymes. The table in Figure 3B shows that the KS test comparing these distributions for 3,4 and 6 step loops have a statistically significant difference (KS test, p-value < 0.05). This is also reflected in the distributions of 3 & 4-loops (Figure 3B right top), and the distributions for feed-forward 3 & 4-loops (FFL 3&4 Figure 3B right bottom), for this last one the p-value is very close to being statistically significant. A measure of the FFL3-loops and FFL4-loops for each node *i* is given by [*MM*^*T*^*M*]_*ii*_ and [*MM*^*T*^*MM*^*T*^]_*ii*_. Furthermore we explored the possibility that the set of nodes defined by localized enzymes has a higher mean loop density than the rest of the nodes. Loop density is defined as [*M*^*n*^]_*ii*_ normalized to the number of connections per node – given by [*MM*^*T*^]_*ii*_ – for reactions catalyzed by localized and non-localized enzymes. We compared the difference in mean loop density in the nodes derived from 189 reactions catalyzed by localized enzymes to nodes derived from 1025 reactions catalyzed by non-localized enzymes to the null hypothesis distribution generated by randomly choosing 10^5^ times 189 reactions in the *E.coli* metabolic network, and calculating each time the mean loop density difference to the remaining 1025 reactions (see diagram Figure 3A right). The table in Figure 3C displays the associated p-values for this null-hypothesis showing a significant higher density of 3 to 6 loops and FF3-4 loops in nodes derived from localized enzymes compared to nodes chosen randomly.

In summary, our analysis shows that localized reactions, while having a higher density of connections among themselves than non-localized ones, have a less rich and more tree-like internal structure. However, these localized reactions have a relatively higher density of feed-forward and feedback loops across the entire network, product of the larger number of motifs they establish with non-localized ones (Additional file 4: Figure S2A red dots). As it might seem paradoxical that localized enzymes form a tree-like structure but also have a higher loop density in the entire network, we decided to explore the nature of loops containing localized and non-localized enzymes. We found that the number of triangles composed of 1 localized enzyme and 2 non-localized enzymes is twice the number of triangles composed of 2 localized and 1 non-localized enzyme. This ratio is 3.6 (statistically significantly different, t-test p-value < 1E-14) when calculated on degree-preserving surrogate networks, where the nodes have the same number of connections as the original network, but higher-order structures are destroyed (see *Methods*). This explains how localized enzymes have a higher loop density as triangles formed by 2 localized and 1 non-localized enzymes are almost twice as many as would be naively expected. The left diagram of Figure 3A summarizes the observed properties of localized enzymes in *E.coli* metabolic network.

### Enzyme localization is dynamic and correlates with strong catalytic activity

In order to understand the role of enzyme localization, we performed a gene ontology (GO) enrichment analysis of the list of localized enzymes. Significantly enriched GO terms related to enzymatic functions that are essential for growth, the top ones being nucleotide, ion, ATP and amino acid binding as well as ubiquinone synthesis (Table 1). Ubiquinone plays a vital role in the electron transport chain and is necessary for normal growth under aerobic conditions [24]. A review of the organization of the amino-acid biosynthetic pathways reveals that 15 out of the 20 pathways have at least one enzyme localized to a cytoplasmic locus and for the 5 amino acids left (alanine, aspartic acid, isoleucine, tyrosine and valine) the pathways consist of only one dedicated enzyme.

**Table 1:** **Table of enriched gene ontology categories**

**Gene ontology enrichment analysis**	**Enzymes**	**Localized enzymes**	**Expected**	**P-values fisher’s exact test**
Nucleotide binding	438	**46**	25	2.11E-05
Manganese ion binding	70	**14**	4	3.08E-05
Magnesium ion binding	170	**23**	10	8.06E-05
ATP binding	427	**43**	25	1.15E-04
Ubiquinone synthesis	18	**6**	1	3.49E-04
Amino acid binding	18	**6**	1	3.49E-04
5′-nucleotidase activity	4	**3**	0.2	7.19E-04
Chemotaxis	22	**6**	1.3	1.16E-03
L-lyxose metabolism	5	**3**	0.3	1.72E-03
Lyase activity	80	**12**	4.6	1.79E-03
Fatty acid biosynthesis	17	**5**	1	2.10E-03

Table of enriched GO categories for *E.coli* enzymes showing GFP localization under exponential growth. First column shows the category, second column the number of enzymes in that category, third column the number of enzymes from the list of 219 localized ones that are in the category, fourth column is the number of enzymes in the category expected by chance and the fifth column is the p-value calculated as the Fisher exact test.

**Figure 4:**
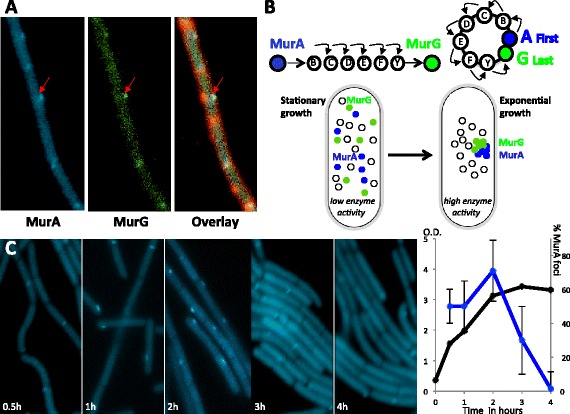
**Localization dynamics of cell wall synthesis enzymes. A**. Red arrow indicates a cell where MurA-CFP (left) and MurG-GFP (middle) colocalize as shown in the overlaid image (right) together with membrane dye (red). **B**. Model for organization of enzymes from the cell wall synthesis pathway going from a diffuse localization during stationary growth to a discrete concentration of MurA and MurG during exponential growth. **C**. *Left images*, Timecourse distribution of enzyme MurA-CFP in *B.subtilis* during exponential and stationary growth. *Right graph*, percentage of cells showing over time localized MurA-CFP (blue, right axis) compared to the culture optical density (black, left axis).

As the significant list of GO terms reflects enzymatic activities necessary for *E.coli* cells growing exponentially, we decided to test whether enzyme localization would vary in different growth conditions. We studied the dynamics of cellular organization in exponential and stationary growth in the bacterium *B. subtilis*. Due to its larger size, *B. subtilis* has emerged as an excellent model organism for bacterial cytology and in particular to study the very well conserved set of cell wall synthesizing enzymes [25]. We fluorescently tagged the cytoplasmically localized set of enzymes MurABCDEFG (see Additional files 5 and 6: Text S7&S8 Plasmids & Strains) and observed that the fluorescent signal from these fusions showed a diffuse localization in the cytoplasm except for the first enzyme MurA-CFP and the last enzyme MurG-GFP (see Figure 4A). In up to 70% of exponentially growing cells from 10 experiments performed in the strain JDB1925, MurA-CFP forms a bright fluorescent spot per cell (Figure 4A&C), localizing principally at septa (39 ± 5% of at least 100 cells), lateral walls (21 ± 5%), poles (29 ± 5%) and in a few cases cytoplasmically (11 ± 5%). In strain JDB2501 MurG-GFP expressed during exponential growth also localized and in strain JDB2840 cells expressing both MurA-CFP/MurG-GFP show co-localization of both proteins (Figure 4A). This shows that the distribution of the cell wall synthesis enzymes in *E.coli* as shown in Additional file 1: Figure S1B is conserved in *B.subtilis* and in both organisms only MurA and MurG show specific localization.

Cells continue to grow exponentially until nutrients become scarce and as their growth rate diminishes they enter a phase of stationary growth. We monitored the transition from exponential to stationary growth by measuring in at least 10 different experiments, optical densities in *B.subtilis* cell cultures strain JDB2840 expressing MurA-CFP (Figure 4C black line in graph) and counted cells showing MurA-CFP localization (Figure 4C blue line in graph). For the first two hours of exponential growth up to 70% of the cells showed MurA-CFP localization, but as cells transitioned to stationary growth the percentage of cells showing MurA-CFP localization plummeted to almost none (Figure 4C images and blue line in graph). Exponentially growing cells divide rapidly and need to synthesize cell wall, hence MurA and MurG enzymes localize when cell wall synthesis is most required. Conversely, as stationary cells grow slower they synthesize less cell wall and MurA and MurG become diffuse in the cytoplasm (see diagram in Figure 4B). This observation sustains the possibility that localization might underlie an enzymatic regulatory strategy where enzymes localize and concentrate in single foci during periods of high catalytic activity and become diffuse during periods of low activity. Localizing the first and last enzymes of a pathway could be an advantageous strategy to restrict the initial and end products to a reduced volume and also putatively concentrate the whole set of reactions (see diagram in Figure 4B) [26].

## Discussion

We studied the sub-cellular cytoplasmic distribution of *E.coli* enzymes in exponentially growing cells from images in a library of GFP-tagged proteins and found that at least 219 of the 857 known *E.coli* enzymes, instead of being diffusively dispersed, show focal localization.

### Enzyme localization is determined by cell’s metabolic state

Fluorescence imaging of *B.subtilis* enzymes from the cytoplasmic part of the cell wall synthesis pathway revealed that their localization can be dynamic. MurA and MurG –the first and the last enzyme of this pathway respectively– concentrate to the same locus during exponential growth and are diffuse during stationary growth (Figure 4). The concentration of these enzymes to a cytoplasmic *lieu* may reflect their active catalytic state, as during exponential growth cell wall is heavily synthesized and its production is low during stationary growth. The enriched GO terms from *E.coli* localizing enzymes also reflect the set of active enzymes in exponentially growing cells as included are amino acid, ATP and ion binding terms, as well as ubiquitone synthesis, all essential during rapid aerobic growth (see Table 1). In support of this idea, it has recently been shown in eukaryotes that in absence of intra-cellular purine [27], adenine or glutamine [28] enzymes of these metabolic pathways form insoluble assemblies and distinct cytoplasmic foci. The formation and dissolution of these punctuate foci is controlled by the absence or presence of nutrients, but the origin of their sub-cellular localization as for the remaining enzymes in this study, has seldom been characterized [29,30].

Reconstruction of the *E.coli* metabolic network also reveals that localized enzymes indicate nodes that play an important role in metabolic activity as they are traversed by more loops (Figure 3). Just like a node with high number of connections, loop number and loop density can be seen as a measure of a higher local flux derived from the network structure. With this in mind, one could hypothesize that in order to rapidly adapt to environmental or cellular changes like fluctuations in enzyme levels, the metabolic network activity is efficiently modified via changes of its local properties. A theoretical study supports this view by stating that local network morphology should be reflected in the temporal propagation –here changes in enzyme localization– of the response to an external perturbation [31]. In this view a specific set of localized enzymes could mirror the current metabolic activity of the network by defining sets of highly active nodes.

### Metabolic adaptability via enzyme localization

Intriguingly, although a considerable subset of enzymes localize, not all do. The temporal study of the cell wall synthesis pathway has shown that only two of its enzymes localize, which was further corroborated for a large set of metabolic pathways as one out of seven reactions localize in *E.coli* growing exponentially (Figure 1B). Why only 25% of enzymes localize in the cell’s cytoplasm and preferentially the first and last?

One answer relies on the highly inter-connected network of localized enzymes organized in a tree-like structure (Figure 3A). This topology effectively interconnects different pathways of *E.coli* metabolism by organizing the metabolic network into sets of pathways bordered and connected via localized enzymes (Figure 3A). Another answer derives from considering two extreme alternatives for the organization of metabolic reactions in the cell cytoplasm; either all enzymes are bound together in a tight biochemical complex, or they diffuse freely without interacting with each other. In the first case, the substrates would be “channeled” within the supra-enzymatic complex; in the second option, the formation for each step of the pathway of a catalytic Michaelis complex between the enzyme-substrate couple and its dissolution after the modification of the substrate would ensure the chemical order of the reaction steps. In a biochemical complex composed of the first and last enzyme of the pathway, intermediary substrates and enzymes of the reaction may still be concentrated around the localized enzymes (see Figure 4B). This observed configuration could conserve the advantages of concentrating substrate and enzyme to enhance the reaction rates [32,33] and regulate spatially the reactions to avoid cross-talk between the myriad chemical reactions that occur simultaneously in a living cell [34]. Further advantages sustaining the strategy of regulating the first and last enzyme of a pathway have been described for transcriptionally sparsely regulated metabolic pathways where it is shown to reduce the cost of protein production while maintaining the ability to rapidly respond to environment changes [21]. It is also supported by the notion that variations in intermediate metabolites in a linear pathway are statistically independent [31] underlying the important strategy of regulating the first and last node.

## Conclusions

In conclusion, we here show for the first time that enzyme localization follows strict rules that could be a measure of *E.coli*’s metabolic state and the topology of the metabolic network while bringing regulatory and catalytic advantages. Localized enzymes in *E.coli* also interconnect metabolic pathways and - as seen for the cell wall synthesis in *B.subtilis* – may organize them spatially in complexes through mechanisms yet to be understood. Finally, regulation of enzyme localization in the cytoplasm could be used in higher-order biota as a general strategy to optimize cell metabolism.

## Methods

### Genobase images (http://ecoli.aist-nara.ac.jp/GB6/search.jsp)

Expression of the cloned ORF is under the control of an IPTG-inducible promoter, which is strictly repressed by lacI^q^ repressor gene product. The strain is without deletion of the corresponding wild-type chromosomal coding DNA sequences (CDSs). Genobase Images were generated using the ASKA library and obtained from *E.coli* cultures growing exponentially and expressing each an enzyme fused C-terminally to a GFP (NO addition of 0.1 mM IPTG). *E.coli* K-12 W3110 strain has 4364 ORFs, 4351 were tagged with a C-terminal GFP fusion and established as plasmid clones as ASKA library. As indicated in the ASKA website, 516 fusions showed GFP focal distribution, 2874 were shown to be around the whole cell, 390 showed membrane distribution, 103 showed both membrane and focal pattern and 355 showed no GFP signal.

### Fluorescence microscopy

For *B.subtilis* cultures growing exponentially in DSM medium at 37°C, 100 μl of cells were taken at designated times. To stain membranes, 0.5 μl of FM4-64 (100 mg/ml) was added to samples just before the cells were collected by centrifugation. The pellet was resuspended in 10 μl PBS, and added to a poly-L-lysine pre-treated coverslip. All microscopy was performed on a Nikon Eclipse 90i with a 100X objective using phase contrast and captured by a Hamamatsu Orca-ER camera using Nikon Elements BR software. Exposure for GFP, CFP and TRITC was 400 ms for all pictures taken.

### Metabolic network analysis

We used the 86 metabolic pathways of *E.coli* defined in KEGG and extracted 1214 unique reactions ordered by their R00000 number (see file Additional file 7: Text S1). Using the KEGG definitions of compounds, reactions and enzymes where each reaction compounds and reversibility/irreversibility are defined (see files Additional file 8: Text S5 for definition of reversible reactions, Additional file 9: Text S4 and Additional file 10: Text S6 for irreversible reactions), we built an asymmetric Boolean square matrix *Mij* of 1214 unique reactions where each reaction is associated to an enzyme and a non-zero matrix value *Mij = 1* indicates that the reaction *i* feeds into reaction *j* by producing a compound used by reaction *j*. We excluded compounds that had more than 60 connections (C00001, H_2_O to C00022, pyruvate) and used the list of localized genes from Genobase defined by gene numbers b0000 (see file Additional file 11: Text S2) to define the 189 localized reactions (see file Additional file 12: Text S3). Scaffold triangulations were calculated using an adapted Matlab code from here. Degree preserving random wiring of network was performed as in [35] using an adapted Matlab code from here.

### Calculating the null hypothesis for the probability of enzyme localization

The probability that the enzyme that catalyzes the first of L reactions in an Elementary Flux Pattern (EFP), given that there are k localized enzymes in that EFP is given by:

$$ P\left(1st\ \mathrm{localized}\left|k,L\right.\right)=\frac{\left(\begin{array}{c}\hfill L-1\hfill \\ {}\hfill k-1\hfill \end{array}\right)}{\left(\begin{array}{c}\hfill L\hfill \\ {}\hfill k\hfill \end{array}\right)}=\frac{k}{L}; $$

If we call *q(k*;*L*) the probability that there are *k* enzymes localized in any position in an EFP of length *L*, then the probability that the first enzyme is localized is

$$ P\left({1}^{\mathrm{st}}\ \mathrm{localized}\left|L\right.\right)={\displaystyle \sum_{k=1}^LP\left({1}^{\mathrm{st}}\ \mathrm{localized}\left|\mathrm{k};\ L\right.\right)q\left(k;L\right)={\displaystyle \sum_{k=1}^L\frac{k}{L}q\left(k;L\right)}} $$

The same expression holds for the probability that the last reaction is catalyzed by a localized enzyme:

$$ P\left(\mathrm{last}\ \mathrm{localized}\left|L\right.\right)={\displaystyle \sum_{k=1}^L\frac{k}{L}q\left(k;L\right)}. $$

The probability that the first and last are localized, given that there are k localized enzymes in an EFP of length L is

$$ P\left({1}^{\mathrm{st}}\ \mathrm{and}\ \mathrm{last}\ \mathrm{localized}\left|k;L\right.\right)=\frac{\left(\begin{array}{c}\hfill L-2\hfill \\ {}\hfill k-2\hfill \end{array}\right)}{\left(\begin{array}{c}\hfill L\hfill \\ {}\hfill k\hfill \end{array}\right)}=\frac{k\left(k-1\right)}{L\left(L-1\right)}; $$

Therefore the probability that the first and last are localized given that the EFP is of length L is

$$ P\left({1}^{\mathrm{st}}\ \mathrm{and}\ \mathrm{last}\ \mathrm{localized}\left|L\right.\right)={\displaystyle \sum_{k=2}^L\frac{k\left(k-1\right)}{L\left(L-1\right)}q\left(k;L\right),} $$

Finally, the probability that the first or the last reaction is localized is equal to:

$$ \begin{array}{c}\hfill P\left({1}^{\mathrm{st}}\ \mathrm{or}\ \mathrm{last}\left|L\right.\right)=P\left({1}^{\mathrm{st}}\left|L\right.\right)+P\left(\mathrm{last}\left|L\right.\right)-P\left({1}^{\mathrm{st}}\mathrm{and}\ \mathrm{last}\left|L\right.\right)\hfill \\ {}\hfill =\frac{2}{L}q\left(1;L\right)+{\displaystyle \sum_{k=2}^L\left[\frac{2k}{L}-\frac{k\left(k-1\right)}{L\left(L-1\right)}\right]q\left(k;L\right)}\hfill \end{array} $$

## Additional files

Additional file 1: Figures S1. A. Images from localized enzymes from the ASKA library, gene names are indicated for each image. B. Images and diagram of localized enzymes from the Cell Wall synthesis pathway. C. Images and diagram of localized enzymes from the leucine pathway. In the diagrams, indicated in green are the reactions for which the associated enzyme localizes and in blue reactions where enzymes are diffuse. Triangle indicates *amino-acids*, square *carbohydrates*, circles for *other metabolites*. Additional file 2: Table S1. List of Elementary Flux Patterns. List of 435 EFPs with at least one enzyme localized. Each line defines a different EFP and the *E.coli* gene numbers b0000 of the enzymes catalyzing the reactions are indicated following the EFP reaction order from left to right; when a gene number is not available, the reaction name is given instead. The list of 219 localized enzymes is indicated in the file Additional file 11: Text S2. Note that some of the EFPs used in the analysis translate into the same gene-set, not repeated here. Additional file 3: Table S2. Table of p-values for localized enzymes. Indicated are the p-values obtained for the probability dependent on varying EFP length (first column) of having the first or last enzyme localized (second column), the first and last enzyme localized (third column). Additional file 4: Figure S2. A. Matrix representing 1214 reactions, dots are present when two reactions are connected via a commonly shared compound but none of the enzymes associated to the reactions are localized (blue), at least one of the enzymes is localized (red), both enzymes are localized (green). B. Each dot represents the number of outcoming reactions plotted in Log-Log scale against the number of incoming reactions for a particular enzyme. Nodes derived from localized enzymes are shown in green, non-localized enzymes in blue. Boxes represent the associated distributions of the number of incoming/outcoming connections per node normalized to its degree, the normalized histograms are *top* for outcoming reactions and *bottom* for incoming reactions, green line is for localized enzymes, blue non-localized. Additional file 5: Text S7. Plasmids&Strains construction. Additional file 6: Text S8. Plasmids&Strains description. Additional file 7: Text S1. All reactions R-numbers for all used reactions. Additional file 8: Text S5. REV Definition of Reversible reactions by compound C-number. The first two columns are the reactants and the last two columns are the products. C000 is a placeholder for the absence of compound. Additional file 9: Text S4. LR Definition of Left-Right reactions by compound C-number. The first two columns are the reactants and the last two columns are the products. C000 is a placeholder for the absence of compound. Additional file 10: Text S6. RL Definition of Right-Left reactions by compound C-number. The first two columns are the reactants and the last two columns are the products. C000 is a placeholder for the absence of compound. Additional file 11: Text S2. Loc genes b-numbers for localized genes. Additional file 12: Text S3. Loc reactions R-numbers for reactions catalyzed by enzymes from genes defined in Text S2_Loc_genes.
