# Supplementary material for: Spatial localization of the first and last enzymes effectively connects active metabolic pathways in bacteria
Source: BMC Syst Biol. 2014 Dec 14;8:131. doi: 10.1186/s12918-014-0131-1 (PMC4279816; doi:10.1186/s12918-014-0131-1)
Supplement: Additional file 1: Figures S1. — A. Images from localized enzymes from the ASKA library, gene names are indicated for each image. B. Images and diagram of localized enzymes from the Cell Wall synthesis pathway. C. Images and diagram of localized enzymes from the leucine pathway. In the diagrams, indicated in green are the reactions for which the associated enzyme localizes and in blue reactions where enzymes are diffuse. Triangle indicates amino-acids, square carbohydrates, circles for other metabolites. [file 12918_2014_131_MOESM1_ESM.pdf]

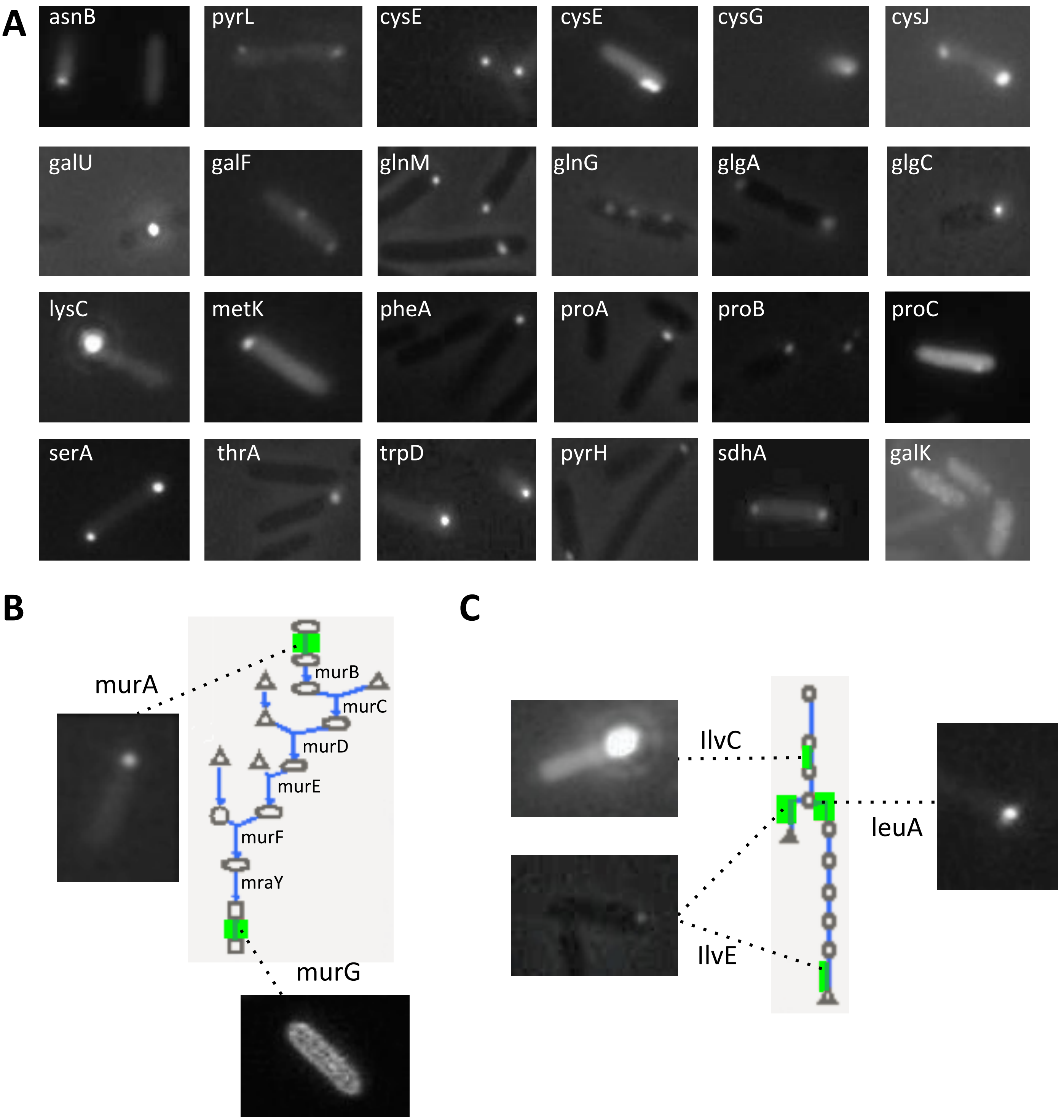

Figures S1 A. Images from localized enzymes from the ASKA library, associated gene names are indicated in the images **B.** Images and associated diagram of localized enzymes from the Cell wall synthesis pathway. **C.** Images Images and associated diagram of localized enzymes from the leucine pathway. In the diagrams, indicated in green are the reactions for which the associated enzyme localizes and in blue reactions where enzymes are diffuse. Triangle indicates *amino-acids*, square *carbohydrates*, circles for *other metabolites.*
