# Supplementary material for: Spatial localization of the first and last enzymes effectively connects active metabolic pathways in bacteria
Source: BMC Syst Biol. 2014 Dec 14;8:131. doi: 10.1186/s12918-014-0131-1 (PMC4279816; doi:10.1186/s12918-014-0131-1)
Supplement: Additional file 4: Figure S2. — A. Matrix representing 1214 reactions, dots are present when two reactions are connected via a commonly shared compound but none of the enzymes associated to the reactions are localized (blue), at least one of the enzymes is localized (red), both enzymes are localized (green). B. Each dot represents the number of outcoming reactions plotted in Log-Log scale against the number of incoming reactions for a particular enzyme. Nodes derived from localized enzymes are shown in green, non-localized enzymes in blue. Boxes represent the associated distributions of the number of incoming/outcoming connections per node normalized to its degree, the normalized histograms are top for outcoming reactions and bottom for incoming reactions, green line is for localized enzymes, blue non-localized. [file 12918_2014_131_MOESM4_ESM.pdf]

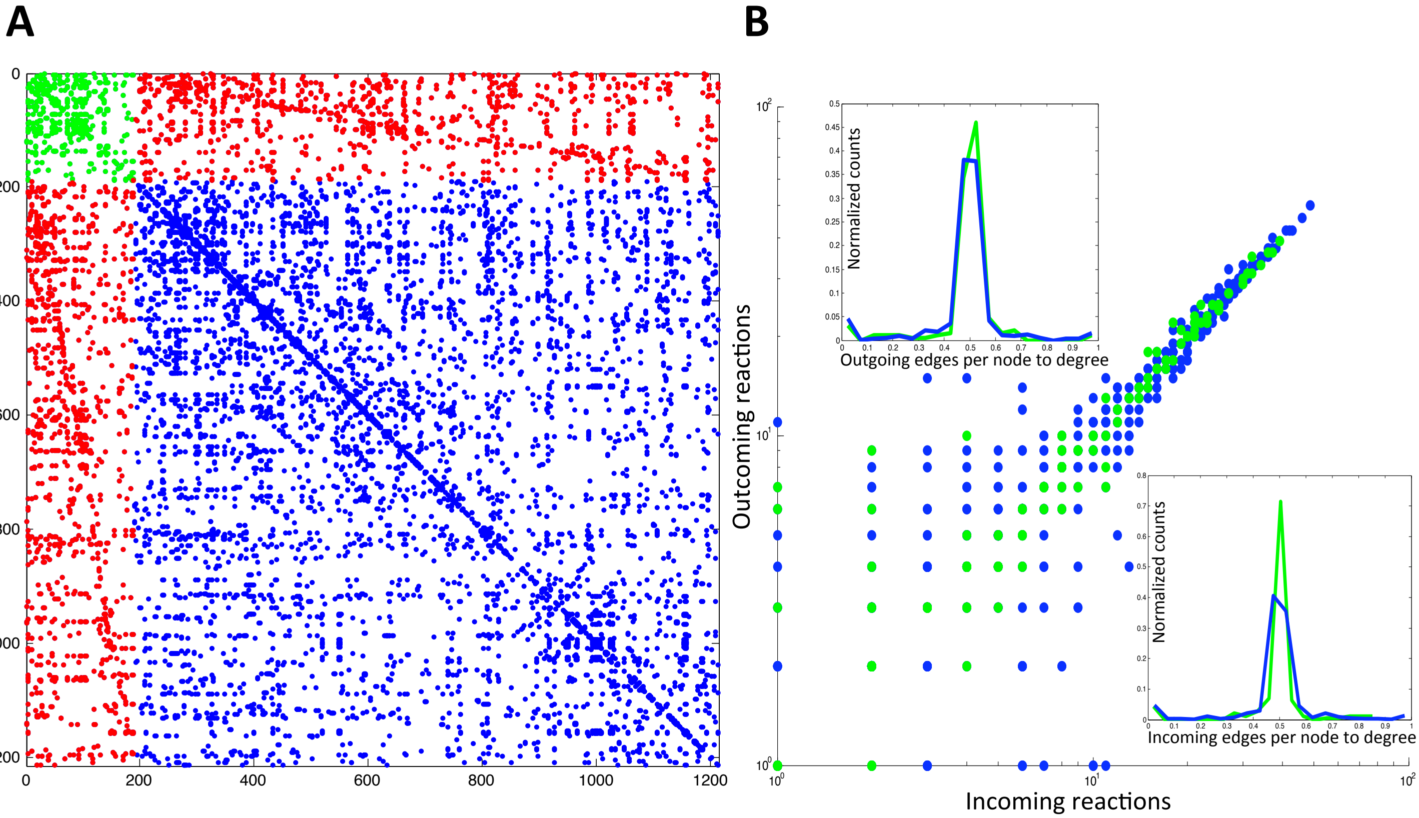

Figure S2 A. Matrix representing 1214 reactions, dots are present when two reactions are connected via a commonly shared compound but none of the enzymes associated to the reactions are localized (blue), at least one of the enzymes is localized (red), both enzymes are Localized (green). **B.** Each dot represents the number of outcoming reactions plotted in Log-Log scale against the number of incoming reactions for a particular enzyme. Nodes derived from localized enzymes are shown in green, non-localized enzymes in blue. Boxes represent the associated distributions of the number of incoming/outcoming connections per node normalized to the degree. The normalized histograms are *top* for outcoming reactions and *bottom* for incoming reactions, green line is for localized enzymes, blue non-localized.
