# Supplementary material for: Spatial localization of the first and last enzymes effectively connects active metabolic pathways in bacteria
Source: BMC Syst Biol. 2014 Dec 14;8:131. doi: 10.1186/s12918-014-0131-1 (PMC4279816; doi:10.1186/s12918-014-0131-1)
Supplement: Additional file 5: Text S7. — Plasmids&Strains construction. [file 12918_2014_131_MOESM5_ESM.pdf]

Bacterial and DNA manipulation

In this article MurAA is referred as MurA for simplicity.

All protein fusions -except MurD and MurE, whose function could not be tested as their genomic loci are in the middle of the operon- were functional.

**Strain**	***Genotype***	**Source**
B. subtilis		
PY79	*wt*	Laboratory stock
JDB1692	*murF::murF-GFP spec*	this work
JDB1920	*murB:murB-CFP spec*	this work
JDB1925	*murAA:murAA-CFP spec*	this work
JDB1944	*murAB:murAB-YFP spec*	this work
JDB1954	*amyE:PspoVE-murC-YFP cm*	this work
JDB2084	*amyE:PspoVE-MurD-YFP cm*	
JDB2085	*amyE:PspoVE-MurE-YFP cm*	
JDB2480	*amyE:Phyperspank-murG cm murG::tet*	Meyer et al (2010)
JDB2501	*amyE::PspoVE-PftsW-murG-GFP spec cm murG::tet*	Meyer et al (2010)
JDB2606	*amyE: PspoVE-PftsW-MurG-GFP cm MurAA-CFP spec*	this work
JDB2840	*amyE: PspoVE-PftsW-MurG-GFP cm MurAA-CFP spec murG::tet*	this work

**Strain**	***Genotype***	**Source**
E. coli	*Plasmids*	
BL21		Laboratory stock
	pAF022	(Fay et al 2010)
	pAF034	this work
	pAF53	(Real et al., 2008)
	pAF73	this work
	pAF118	this work
	pAF129	this work
	pAF239	(Fay et al 2010)

pAF366	this work
pAF367	this work
pDG1662	(Guerout-Fleury 1996)
pKM10	(Fay et al 2010)
pMR1	this work
pMR3	this work
pMR4	this work
pMR6	this work
pMR7	this work
pMR8	this work
pMR11	this work
pMR16	this work
pMR29	this work
pMR41	this work

Plasmid construction

pMR3 A PCR product from PY79 genomic DNA containing 506bp of 3'*murAB* using 5' primer opm33 and 3' primer opm34 was digested with the restriction enzymes *EcoR*I and *Xho*I and inserted into plasmid *pKL147* containing CFP digested with *EcoR*I and *Xho*I.

pMR4 A PCR product from PY79 genomic DNA containing ~500bp of 3'*murB* using 5' primer opm25 and 3' primer opm26 was digested with the restriction enzymes *EcoR*I and *Xho*I and inserted into plasmid *pKL147* containing YFP digested with *EcoR*I and *Xho*I.

pMR6 A PCR product from PY79 genomic DNA containing *murC* using 5' primer opm76 and 3' primer opm90 was digested with the restriction enzymes *Spe*I and *BamH*I and inserted into plasmid *pKM44* digested with *Spe*I and *BamH*I. A PCR product from *pKL147* containing YFP using 5' primer aof241 and 3' primer

aof253 was digested with the restriction enzymes *BamH*I and *Bgl*II and inserted into *pKM44-murC* cut with *BamHI.*

pMR7 A PCR product from PY79 genomic DNA containing *murD* using 5' primer opm77 and 3' primer opm71 was digested with the restriction enzymes *Spe*I and *BamH*I and inserted into plasmid *pKM44* containing CFP digested with *Spe*I and *BamH*I.

pMR8 A PCR product from PY79 genomic DNA containing *murE* using 5' primer opm42 and 3' primer opm65 was digested with the restriction enzymes *Spe*I and *BamH*I and inserted into plasmid *pKM44* containing YFP digested with *Spe*I and *BamH*I.

pMR11 A PCR product from PY79 genomic DNA containing ~500bp of 3'*murF* using 5' primer ojd197 and 3' primer ojd198 was digested with the restriction enzymes *EcoR*I and *Xho*I and inserted into plasmid *pKL147* containing YFP digested with *EcoR*I and *Xho*I.

pMR16 A PCR product from PY79 genomic DNA containing ~500bp of 3'*murAA* using 5' primer opm31 and 3' primer opm154 was digested with the restriction enzymes *EcoR*I and *Xho*I and inserted into plasmid pKL147 digested with *EcoR*I and *Xho*I.

pMR29 pKM59 was digested with the restriction enzymes *Spe*I and *BamH*I and inserted into plasmid pAF53 cut with *Spe*I and *BamH*I.

pMR41 A PCR product from PY79 genomic DNA containing ~500bp of 3'*murF* using 5' primer ojd197 and 3' primer ojd198 was digested with the restriction enzymes *EcoR*I and *Xho*I and inserted into plasmid pKL147-CFP digested with *EcoR*I and *Xho*I.

pAF073 A PCR with a new region with added sites *Spe*I, *Nhe*I was amplified using 5' primer aof161 and 3' primer aof162 from pDG1662 and digested with the restriction enzymes *Aat*II and *BamH*I and ligated to pDG1662 cut with *Aat*II and *BamH*I.

pAF129 A PCR product from PY79 genomic DNA containing *murG* using 5' primer aof328 and 3' primer aof329 was digested with the restriction enzymes *Nhe*I and *Sph*I and inserted into plasmid pDR111 digested with *Nhe*I and *Sph*I.

pAF366 A PCR from PY79 genomic DNA containing *murG* knock-out front flank using 5' primer aof336 and 3' primer aof337 was digested with the restriction enzymes *Spe*I and *BamH*I and inserted into pAF073 cut with *Spe*I and *BamH*I.

pAF367 A PCR from PY79 genomic DNA containing *murG* knock-out back flank with 5' primer afo715 and 3' primer afo716 was digested with restriction enzymes *Sal*I and *Bgl*II then inserted into pAF366 cut with *Sal*I and *Bgl*II.

pKM10 A PCR product from PY79 genomic DNA containing 506bp of 3'*murG* using 5' primer ojd010 and 3' primer ojd023 was digested with the restriction enzymes *EcoR*I and *Xho*I and inserted into plasmid pKL147 containing GFP digested with *EcoR*I and *Xho*I.

Strain construction

JDB1692 (*murF:murF-YFP spec*) transform *pMR11* into PY79 select for spec^R.

JDB1911 *(amyE:Phyperspank-murG cm*) transform *pAF129* into PY79 select for cm^R, and screen amy-.

JDB1920 (*murB:murB-CFP spec*) transform *pMR4* into PY79 select for spec^R.

JDB1925 (*muAA:murAA-CFP spec*) transform *pMR*16 into PY79 select for spec^R.

JDB1944 (*muAB:murAB-YFP spec*) transform *pMR3* into PY79 select for spec^R.

JDB1954 (*amyE:PspoVE-murC-YFP cm*) transform *pMR6* into PY79 select for cm^R, screen for amy-.

JDB2084 (*amyE:PspoVE-MurD-YFP cm*) transform *pMR7* in PY79 select for cm^R, screen for amy-.

JDB2085 (*amyE:PspoVE-MurE-YFP cm*) transform *pMR8* in PY79 select for cm^R, screen for amy-.

JDB2480 (*amyE:Phyperspank-murG cm murG::tet)* transform *pAF367* into JDB1911 select for tet^R screen for amy-, cm^R.

JDB2498 (*amyE::PspoVE-PftsW-murG cm*) transform *pMR29* into JDB2537 select for cm^R, screen for sac-, tet^R, kan^R.

JDB2499 (*amyE::PspoVE-PftsW-murG-gfp spec cm*) transform *pKM10* into JDB2498 select for spec^R, screen for amy-, cm^R.

JDB2501 (*amyE::PspoVE-PftsW-murG-gfp spec cm murG::tet*) transform DNA from JDB2480 into JDB2499 select for tet, screen for spec^R, cm^R, amy-.

JDB2606 (*amyE: PspoVE-PftsW-MurG-GFP cm MurAA-CFP spec*)

JDB2840 (*amyE: PspoVE-PftsW-MurG-GFP cm MurAA-CFP spec murG::tet)*
