# Supplementary material for: Spatial localization of the first and last enzymes effectively connects active metabolic pathways in bacteria
Source: BMC Syst Biol. 2014 Dec 14;8:131. doi: 10.1186/s12918-014-0131-1 (PMC4279816; doi:10.1186/s12918-014-0131-1)
Supplement: Additional file 6: Text S8. — Plasmids&Strains description. [file 12918_2014_131_MOESM6_ESM.pdf]

Strain	*Genotype*	Source	Details of construction
B. subtilis			
PY79	*wt*	Laboratory stock	
JDB1692	*murF::murF-GFP spec*	this work	transform pMR11 in PY79 select spec
JDB1920	*murB:murB-CFP spec*	this work	transform pMR4 in PY79 select spec
JDB1925	*murAA:murAA-CFP spec*	this work	transform pMR1 in PY79 select spec
JDB1944	*murAB:murAB-YFP spec*	this work	transform pMR3 in PY79 select spec
JDB1954	*amyE:PspoVE-murC-YFP cm*	this work	transform pMR6 in PY79 select cm
JDB2084	*amyE:PspoVE-MurD-YFP cm*	this work	transform pMR7 in PY79 select cm
JDB2085	*amyE:PspoVE-MurE-YFP cm*	this work	transform pMR8 in PY79 select cm
JDB2480	*amyE:Phyperspank-murG cm murG::tet*	Meyer et al (2010)	
JDB2501	*amyE::PspoVE-PftsW-murG-GFP spec cm murG::tet*	Meyer et al (2010)	transform JDB2480 in JDB2499 select tet
JDB2606	*amyE: PspoVE-PftsW-MurG-GFP cm MurAA-CFP spec*	this work	transform JDB2499 in JDB1925 select cm
JDB2840	*amyE: PspoVE-PftsW-MurG-GFP cm MurAA-CFP spec murG::tet*	this work	transform JDB2480 in JDB2606 select tet

Strain	*Genotype*	Source
E. coli	*Plasmids*	
BL21		Laboratory stock
	pAF022	(Fay et al 2010)
	pAF034	this work
	pAF53	(Real et al., 2008)
	pAF73	this work
	pAF118	this work
	pAF129	this work
	pAF239	(Fay et al 2010)
	pAF366	this work
	pAF367	this work
	pDG1662	(Guerout-Fleury 1996)
	pKM10	(Fay et al 2010)

	pMR1	this work
	pMR3	this work
	pMR4	this work
	pMR6	this work
	pMR7	this work
	pMR8	this work
	pMR11	this work
	pMR16	this work
	pMR29	this work
	pMR41	this work

Plasmids

For B. subtilis integration

	**Purpose**	**Content**
pMR1	3'MurG in pKL147-YFP	
pMR3	3'MurAB in pKL147-CFP	
pMR4	3'MurB in pKL147-YFP	
pMR6	MurC-CFP in pKM44	
pMR7	MurD-YFP in pKM44	
pMR8	MuED-YFP in pKM44	
pMR11	3'MurF in pKL147-YFP	
pKM10	PspoVE-PftsW in pkm44	
pKM59	pkm44-MurG	
pAF34	*amyE::PspoVE-murG-flag Cm*	
pAF44	amyE::tet, spc amp	
pAF53	PspoVE-PftsW	
pAF73	pDG1662 with added SpeI, NheI sites	
pAF118	PspoVE-murG-flag at amyE	
pAF129	Phyperspank-MurG	
pAF239	PspoVE-secY(1,6)-flag at amyE	
pAF366	murGFF-tet-amyEbackflank	
pAF367	MurG deletion	

oligos

AFO241	5''GFP-BglII	(Fay et al 2010)
AFO253	3'GFP-HindIII-BamHI	(Fay et al 2010)
opm25	MurB3'EcoRI	actcgaattctttatatgaatgccggcgctcacg
opm26	MurB5'XhoI	agtcctcgaggcgatttccgccgatgatttcaacc
opm31	MurAA5'EcoRI	gtc gcg aga tct cta ttt gta tag ttc atc cat gc
opm32	MurAA3'XhoI	GATAAGCTTGGATCCGGTTCCGGATGATGAAG
opm33	MurAB5'EcoRI	actcgaattctttatatgaatgccggcgctcacg
opm34	MurAB3'XhoI	agtcctcgaggcgatttccgccgatgatttcaacc
opm71	MurD3'BamHI	gatcgaattcgagatgggtgtaacaattaaggatgaagg
opm76	MurC5'SpeI	agtcctcgagtgcatttaagtcagaaacgacttctttattc
opm77	MurD5'SpeI	actcgaattcaacggcaaagctgagagaaatgggc
opm90	MurC3'BamHI	agtcctcgagtgaattttgaagctgttctatttcttcg
